# Theory and Application of the Information Bottleneck Method

**DOI:** 10.3390/e26030187

**Published:** 2024-02-22

**Authors:** Jan Lewandowsky, Gerhard Bauch

**Affiliations:** 1 Fraunhofer Institute for Communication, Information Processing and Ergonomics, Fraunhoferstraße 20, 53343 Wachtberg, Germany; 2 Institute of Communications, Hamburg University of Technology, Eißendorfer Straße 40, 21073 Hamburg, Germany; bauch@tuhh.de

In 1999, Naftali Tishby et al. introduced a powerful information theoretical framework called the information bottleneck method [1]. Conceptually, the aim of this method was to obtain a compressed representation T of an observed random variable Y by mapping Y onto T. In this setup, compression is caused by the rate–distortion concept of minimizing the mutual information I(Y;T). The plain minimization of I(Y;T), however, would destroy all information stored in Y. While classical rate–distortion theory defines limits on a distortion measure, typically with respect to the observation Y, the information bottleneck method introduces the concept of relevant information. Therefore, the fundamental idea of the information bottleneck method is to determine which information should be preserved under the invoked compression. This relevant information is defined through another random variable X. Hence, preserving the mutual information I(X;T) while minimizing I(Y;T) is the fundamental principle of the information bottleneck method.

The seemingly theoretical setup was proven to be very useful for various practical applications over the last few years. Its utilization evolved and covers too many different fields to list them here exhaustively. Examples include machine learning, deep learning, neuroscience, multimedia and image processing, data processing, source coding, channel coding and information processing. In addition, the theoretical backgrounds of the method, its generalizations and algorithms to find optimum mappings of Y onto T have become fruitful research topics. It should be noted that similar problems have been previously treated by Witsenhausen, Wyner and Ziv in the 1970s. However, this was on a rather abstract level and the information bottleneck with the involved mutual information measures as presented by Tishby and his colleagues was not as clearly carved out.

Today, more than 20 years after the publication of [1], researchers still conduct very promising research on the theory and the applications of the information bottleneck method in various disciplines of science and engineering. In light of this, we initiated a Special Issue to enrich the literature with topical theoretical and applied works oriented around the information bottleneck method. Therefore, we encouraged authors to submit their latest results on the theory and the applications of the information bottleneck method. We intentionally did not narrow the scope to a certain field of science or engineering. As a result, our Special Issue impressively illustrates the extensive theory and broad applicability of the information bottleneck framework.

In the following, we provide an overview of the published articles:

- Motivated by the information bottleneck and information-distortion systems, Parker and Dimitrov study the mathematical structure of information-based distortion-annealing problems in contribution 1. They investigate the bifurcations of solutions of certain degenerate constrained optimization problems related to the information bottleneck setup. Similarly, contribution 1 contributes to characterizing the local bifurcation structure of information bottleneck-type problems.
- Agmon leverages the information bottleneck’s relations with the rate–distortion theory to provide deep insights into its general solution structure in contribution 2. Sub-optimal solutions are seen to collide or exchange optimality at bifurcations of the rate–information curve. By exploiting the dynamics of the optimal trade-off curve, a means to classify and handle bifurcations is presented. This understanding of bifurcations is used to propose a novel and surprisingly accurate numerical information bottleneck algorithm.
- Charvin, Volpi and Polani investigate the extent to which one can use existing compressed information bottleneck representations to produce new ones with a different granularity in contribution 3. First, they consider the notion of successive refinement, where no information needs to be discarded for this transition. For some specific information bottleneck problems, they derive successive refinability analytically and provide a tool to investigate it for discrete variables. Going further, they also quantify the loss of information optimality induced by several-stage processing in information bottleneck setups.
- Dikshtein, Ordentlich and Shamai introduce and study the double-sided information bottleneck problem in contribution 4, which is closely related to the biclustering domain. For jointly Gaussian and doubly symmetric binary sources in the double-sided information bottleneck setup, they provide insights on optimum solutions. They also explore a Blahut–Arimoto-like alternating maximization algorithm to find solutions for double-sided information bottleneck problems.
- Deng and Jia use the information bottleneck concept to deal with out-of-distribution generalization for classification tasks in contribution 5. In this context, they analyze failure situations of the information bottleneck invariant risk minimization principle and propose a new method, termed the counterfactual supervision-based information bottleneck, to overcome them. The effectiveness of their method is demonstrated empirically.
- Lyu, Aminian and Rodrigues use information bottleneck-inspired techniques to investigate the learning process of neural networks in contribution 6. They argue that the mutual information measures involved in the information bottleneck setup are difficult to estimate in this context. Therefore, they replace them with more tractable ones, i.e., the mean-squared error and the cross entropy. The resulting information bottleneck-inspired principle is used to study the learning dynamics of neural networks.
- Moldoveanu and Zaidi study distributed inference and learning over networks in contribution 7. They develop a framework to combine the information of observed features in a distributed information bottleneck setup with distributed observed nodes and a fusion node that conduct inference. Their experiments underline the advantages of their proposed scheme with respect to other distributed learning techniques.
- Steiner, Aminu and Kühn consider the optimization of distributed source coding in sensor networks in contribution 8. They investigate communication protocols in an extension of the so-called “extended chief executive officer problem setup”. In this extension, the involved sensor nodes are allowed to communicate. The sensor nodes are optimized greedily and it is shown that their cooperation improves the performance significantly.
- Toledo, Venezian and Slonim revisit the sequential information information bottleneck (sIB) algorithm in contribution 9. Implementation aspects are discussed and the performance of their optimized information bottleneck algorithm is evaluated. The proposed implementation provides a trade-off between quality and speed that outperforms the considered reference algorithms. The novel sIB implementation is publicly available to ease further research on the information bottleneck method.
- Monsees, Griebel, Herrmann, Wübben, Dekorsy and Wehn study quantized decoders for low-density parity-check codes which are designed using the information bottleneck principle of maximizing the preserved relevant information under quantization in contribution 10. Such decoders allow for coarse quantization with minimum performance losses. A novel criterion for the required bit resolution in reconstruction–computation–quantization decoders is derived. Moreover, a comparison with a min-sum decoder implementation for throughput towards 1 Tb/s in fully depleted silicon-on-insulator technology is carried out.
- Contribution 11 describes the application of the information bottleneck method to quantized signal processing problems in communication receivers. For this purpose, contribution 11 summarizes recent ideas from various works to use the method for low-complexity quantized channel decoding, detection and channel estimation. In addition, novel results on a strongly quantized receiver chain, including channel estimation, detection and channel decoding, illustrate the ability to achieve optimum performance despite strong quantization with the proposed information bottleneck receiver design.

We would like to thank all of the aforementioned authors for their outstanding contributions to this Special Issue. We are also especially grateful to the reviewers. The articles in this Special Issue illustrate the current, highly versatile research directions related to the information bottleneck method, from relatively mathematical concepts and theories to their applications in practical engineering. We believe that research on the information bottleneck method will continue to play a very important role in the future. We look forward to further research work related to this fascinating method.

## List of Contributions

1. Parker, A.E.; Dimitrov, A.G. Symmetry-Breaking Bifurcations of the Information Bottleneck and Related Problems. *Entropy* **2022**, *24*, 1231.
2. Agmon, S. The Information Bottleneck’s Ordinary Differential Equation: First-Order Root-Tracking for the IB. *Entropy* **2023**, *25*, 1370.
3. Charvin, H.; Volpi, N.C.; Polani, D. Exact and Soft Successive Refinement of the Information Bottleneck *Entropy* **2023**, *25*, 1355.
4. Dikshtein, M.; Ordentlich, O.; Shamai, S. The Double-Sided Information Bottleneck Function. *Entropy* **2022**, *24*, 1321.
5. Deng, B.; Jia, K. Counterfactual Supervision-Based Information Bottleneck for Out-of-Distribution Generalization. *Entropy* **2023**, *25*, 193.
6. Lyu, Z.; Aminian, G.; Rodrigues, M.R.D. On Neural Networks Fitting, Compression, and Generalization Behavior via Information-Bottleneck-like Approaches. *Entropy* **2023**, *25*, 1063.
7. Moldoveanu, M.; Zaidi, A. In-Network Learning: Distributed Training and Inference in Networks. *Entropy* **2023**, *25*, 920.
8. Steiner, S.; Aminu, A.D.; Kuehn, V. Distributed Quantization for Partially Cooperating Sensors Using the Information Bottleneck Method. *Entropy* **2022**, *24*, 438.
9. Toledo, A.; Venezian, E.; Slonim, N. Revisiting Sequential Information Bottleneck: New Implementation and Evaluation. *Entropy* **2022**, *24*, 1132.
10. Monsees, T.; Griebel, O.; Herrmann, M.; Wübben, D.; Dekorsy, A.; Wehn, N. Minimum-Integer Computation Finite Alphabet Message Passing Decoder: From Theory to Decoder Implementations towards 1 Tb/s. *Entropy* **2022**, *24*, 1452.
11. Lewandowsky, J.; Bauch, G.; Stark, M. Information Bottleneck Signal Processing and Learning to Maximize Relevant Information for Communication Receivers. *Entropy* **2022**, *24*, 972.
